# Development and Validation of a Deep Learning Model to Quantify Glomerulosclerosis in Kidney Biopsy Specimens

**DOI:** 10.1001/jamanetworkopen.2020.30939

**Published:** 2021-01-20

**Authors:** Jon N. Marsh, Ta-Chiang Liu, Parker C. Wilson, S. Joshua Swamidass, Joseph P. Gaut

**Affiliations:** 1 Department of Pathology and Immunology, Washington University School of Medicine in St Louis, St Louis, Missouri; 2 Institute for Informatics (I^2^), Washington University School of Medicine in St Louis, St Louis, Missouri; 3 Department of Medicine, Washington University School of Medicine in St Louis, St Louis, Missouri

## Abstract

**Importance:**

A chronic shortage of donor kidneys is compounded by a high discard rate, and this rate is directly associated with biopsy specimen evaluation, which shows poor reproducibility among pathologists. A deep learning algorithm for measuring percent global glomerulosclerosis (an important predictor of outcome) on images of kidney biopsy specimens could enable pathologists to more reproducibly and accurately quantify percent global glomerulosclerosis, potentially saving organs that would have been discarded.

**Objective:**

To compare the performances of pathologists with a deep learning model on quantification of percent global glomerulosclerosis in whole-slide images of donor kidney biopsy specimens, and to determine the potential benefit of a deep learning model on organ discard rates.

**Design, Setting, and Participants:**

This prognostic study used whole-slide images acquired from 98 hematoxylin-eosin–stained frozen and 51 permanent donor biopsy specimen sections retrieved from 83 kidneys. Serial annotation by 3 board-certified pathologists served as ground truth for model training and for evaluation. Images of kidney biopsy specimens were obtained from the Washington University database (retrieved between June 2015 and June 2017). Cases were selected randomly from a database of more than 1000 cases to include biopsy specimens representing an equitable distribution within 0% to 5%, 6% to 10%, 11% to 15%, 16% to 20%, and more than 20% global glomerulosclerosis.

**Main Outcomes and Measures:**

Correlation coefficient (*r*) and root-mean-square error (RMSE) with respect to annotations were computed for cross-validated model predictions and on-call pathologists’ estimates of percent global glomerulosclerosis when using individual and pooled slide results. Data were analyzed from March 2018 to August 2020.

**Results:**

The cross-validated model results of section images retrieved from 83 donor kidneys showed higher correlation with annotations (*r* = 0.916; 95% CI, 0.886-0.939) than on-call pathologists (*r* = 0.884; 95% CI, 0.825-0.923) that was enhanced when pooling glomeruli counts from multiple levels (*r* = 0.933; 95% CI, 0.898-0.956). Model prediction error for single levels (RMSE, 5.631; 95% CI, 4.735-6.517) was 14% lower than on-call pathologists (RMSE, 6.523; 95% CI, 5.191-7.783), improving to 22% with multiple levels (RMSE, 5.094; 95% CI, 3.972-6.301). The model decreased the likelihood of unnecessary organ discard by 37% compared with pathologists.

**Conclusions and Relevance:**

The findings of this prognostic study suggest that this deep learning model provided a scalable and robust method to quantify percent global glomerulosclerosis in whole-slide images of donor kidneys. The model performance improved by analyzing multiple levels of a section, surpassing the capacity of pathologists in the time-sensitive setting of examining donor biopsy specimens. The results indicate the potential of a deep learning model to prevent erroneous donor organ discard.

## Key Points

**Question:**

Can a deep neural network decrease likelihood of unnecessary donor kidney discard by precisely quantifying percent global glomerulosclerosis on whole-slide images of hematoxylin-eosin–stained biopsy specimens?

**Findings:**

In this prognostic study of 83 donor kidneys, a deep neural network segmented normal and globally sclerotic glomeruli in whole-slide images to quantify percent global glomerulosclerosis with higher performance than pathologists. Model accuracy further increased by pooling multiple sections, resulting in decreased likelihood of erroneous organ discard by 37%.

**Meaning:**

This study’s findings suggest that deep learning methods may help prevent erroneous organ discard by performing beyond the capacity of pathologists in biopsy specimen examination.

## Teaser

This prognostic study compares performances between pathologists and a deep learning model to quantify percent global glomerulosclerosis on images of kidney biopsy specimens and assesses the potential benefit of the model on donor kidney discard rates.

## Introduction

More than 100 000 patients are currently waiting for a kidney transplant.^1^ Despite the growing need, between 17% and 20% of kidneys recovered for transplant are discarded.^2,3,4^ With organ shortage and increasing demand for kidney transplants, there is an urgent need to decrease unnecessary organ discard.^3^

The biopsy result is reported as the most important factor in the decision to use or discard a donor kidney.^5^ Numerous investigations have linked chronic damage in donor kidney biopsy specimens with transplant outcomes.^6,7,8,9,10,11,12^ A level of 20% global glomerulosclerosis is frequently used as a cut point in the decision to transplant and is a major factor underlying why the biopsy result is the most common reason an organ is rejected for transplantation in the United States.^4^

Recent studies indicate that acceptable kidneys are being discarded because of variable and inconsistent donor biopsy specimen interpretation.^3,13,14^ Even a seemingly straightforward metric such as the percent global glomerulosclerosis is subject to significant human variation.^15,16,17^ Freezing artifacts, lack of subspecialty expertise, inadequate sampling, and the time-sensitive nature of these evaluations all contribute to human errors.

Recently, deep learning (DL) has shown potential to improve reproducibility and accuracy in histopathologic examination.^18,19,20,21,22,23,24,25,26^ Prior studies from other laboratories have used DL approaches for automated detection of nonsclerotic and globally sclerotic glomeruli.^27,28,29,30,31^ However, these techniques rely on special stains, such as periodic acid–Schiff or Masson trichrome stains, that are impractical in the time-sensitive setting of frozen sections. Previous work from members of our group describes the only reported results, to our knowledge, showing high performance for automated quantitation of percent global glomerulosclerosis using whole-slide images (WSIs) of hematoxylin-eosin–stained frozen sections.

We hypothesize that a DL approach to examination of donor kidney biopsy specimens will outperform human pathologists in evaluating percent global glomerulosclerosis, and that further enhancement will be enabled by examining multiple levels of section. This increased tissue sampling is hypothesized to decrease the likelihood of unnecessary organ discard and will address the question of whether DL techniques are associated with making a substantive increase in the available donor organ pool.

## Methods

This study followed the Transparent Reporting of a Multivariable Prediction Model for Individual Prognosis or Diagnosis (TRIPOD) reporting guideline for diagnostic and prognostic studies. This study was reviewed and approved by the Washington University institutional review board, which also waived the need for obtaining informed patient consent because this study used only nonidentifiable biospecimens from an existing data set.

### Data Collection

The WSIs were acquired from deceased donor biopsy specimens—98 hematoxylin-eosin–stained frozen sections and 51 permanent sections—retrieved from a total of 83 kidneys by using both wedge and needle samples. Frozen-section biopsy specimens and permanent-section biopsy specimens were obtained from different kidneys. Of 83 specimens, 62 had at least 2 levels of section. Biopsy specimen images from the Washington University database originated from Gift of Life Michigan (retrieved between August 2015 and November 2016 using a Sakura scanner; magnification, ×20) and Washington University (retrieved between June 2015 and June 2017 via Mid-America Transplant using an Aperio Scanscope CS scanner; magnification, ×20). Any deceased organ donor who presented between these dates and underwent a kidney biopsy for digital intraoperative pathologic examination was eligible for this study. The demographic characteristics and clinical features of donors were unknown to the investigators. All scans were converted from SVS to TIFF format at full resolution (0.5 μm/pixel). Image sizes ranged from 105 megapixels to 1448 megapixels.

### Data Annotation

Slides were first annotated for nonsclerotic and sclerotic glomeruli by a board-certified expert kidney pathologist (P.W. or J.P.G.), revised by a second board-certified pathologist (T.C.L.) with experience interpreting donor kidney biopsy specimens, and followed by a final revision by another board-certified expert kidney pathologist (P.C.W. or J.P.G.). The final revised annotations served as ground truth (ie, the gold standard) for model training and evaluation. Typical variability in glomeruli counts with each revision is illustrated in eFigure 1 in the Supplement. An in-house plug-in written for Fiji^32^ was used to manually outline and classify glomeruli on each WSI to generate pixelwise label masks of glomerulus regions at the same resolution as the parent WSI. Glomeruli were classified as either globally sclerotic (defined as sclerosis involving the entire glomerular tuft, including obsolescent, solidified, and disappearing global glomerulosclerosis) or nonglobally sclerotic. All other areas were grouped together and labeled tubulointerstitium. A total of 1544 globally sclerosed and 6914 nonglobally sclerosed glomeruli were labeled in 149 separate images. The biopsy specimens exhibited a wide range of percent global glomerulosclerosis (0%-77%). The mean (SD) number of glomeruli per slide was 57 (31).

### DL Model Architecture

The DL model used in this study was a fully convolutional neural network based on the VGG16 architecture^33^ described in previous work, which included a member of our group.^34^ In brief, data were input to the pretrained VGG16 base network with weights frozen below the bottleneck (ie, immediately prior to the densely connected classification layers). The VGG16 densely connected classification layers were replaced with 5 fully convolutional layers with trainable weights. Use of a fully convolutional architecture through the entire network enabled an “image to image” transformation, rather than an “image to label” transformation, for each input image patch, the latter of which is an approach that is less accurate and much more computationally expensive.^34^ The fully convolutional model generated downsampled pixel maps registered to the input image patch, giving the probability that each output pixel was tubulointerstitium, nonglobally sclerosed glomerulus, or globally sclerosed glomerulus.

### Training Parameters

Images were diced into 2048 × 2048-pixel (1024 × 1024 μm) partially overlapping image patches (stride, 1664 pixels or 838 μm) for training input. Patches were selected for training by randomly sampling from the entire pool of image patches (approximately 6500 patches in each cross-validation training set, the length of a single epoch). Input patches were randomly flipped or rotated (by 0°, 90°, 180°, or 270°), yielding 8-fold augmentation of training data for a total of approximately 52 000 possible training patches in the sampling pool. Training was performed using TensorFlow by minimizing categorical cross-entropy loss, weighted classwise using a ratio for sclerosed to nonsclerosed to tubulointerstitial categories of 10:5:1 to compensate for class imbalance. Stochastic gradient descent optimization was used with a cyclic learning rate between 1e^−4^ and 1e^−2^ and a batch size of 4 for 15 epochs.

### Cross-Validation

The model was trained and tested in 10-fold cross-validation, where 10% of the WSIs were withheld from training in each fold, and the resulting model (trained on the remaining 90% of data) was used to generate predictions on the withheld WSIs. Images from different levels of the same kidney were always held out together. No information from a test set of a cross-validation fold was used to inform training of the corresponding fold. Predictions for withheld slides were generated patchwise according to the image dicing scheme described above (ie, 2048 × 2048–pixel patches with 1664-pixel stride), and the results were reassembled to produce output probability maps for entire WSIs.

### Postprocessing

A standard laplacian of gaussian blob detection algorithm, well-suited to identify circular regions of high image intensity at multiple scales,^35^ was used to localize individual glomeruli from the probability maps. Percent global glomerulosclerosis was computed by the formula 100 × *S*/*N*, where *S* is the number of globally sclerosed glomeruli and *N* is the total glomeruli count.

### Statistical Analysis

Pixelwise agreement between annotation and prediction probability maps was quantified via the Dice coefficient and the intersection over union metric, computed in aggregate for all pixels in each output label. Glomeruli counts were obtained after blob detection processing on sclerosed and nonsclerosed probability map channels. Percent global glomerulosclerosis was computed from these counts for individual images, and for individual kidneys, by pooling counts for all levels (typically 2) associated with each kidney. Glomeruli counts were compared against annotation ground truth, with accuracy assessed by Pearson correlation coefficient *r* and root-mean-square error (RMSE). Corresponding quantities for percent global glomerulosclerosis were computed for on-call pathologists’ estimates, and those values were compared with the model’s performance.

Categorization of kidneys as “acceptable” for transplant or “rejected” was determined at 20% global glomerulosclerosis, a commonly used cut point in current clinical practice based on historical data. An F1 score was computed as a function of correctly discriminating whether a sample was over or under the 20% cut point with respect to ground truth annotations. Cohen κ coefficient (an indicator of agreement between raters) was also computed for the model’s and on-call pathologists’ discrimination at the 20% cut point, as compared with ground truth annotation and with each other.

Because the definition of global glomerulosclerosis is naturally expressed as the mean of a beta distribution given by parameters *S* (number of globally sclerosed glomeruli) and (*N* − *S*) (number of nonglobally sclerosed glomeruli), it was used to compute 95% prediction intervals that serve as an indicator of output precision. A 2-sided *P* < .05 was considered statistically significant. All statistical analyses were conducted from March 2018 to August 2020 with the Python packages scikit-learn, version 0.22.1, and SciPy.stats, version 1.4.1.

## Results

### Output Visualization

**Figure 1.**
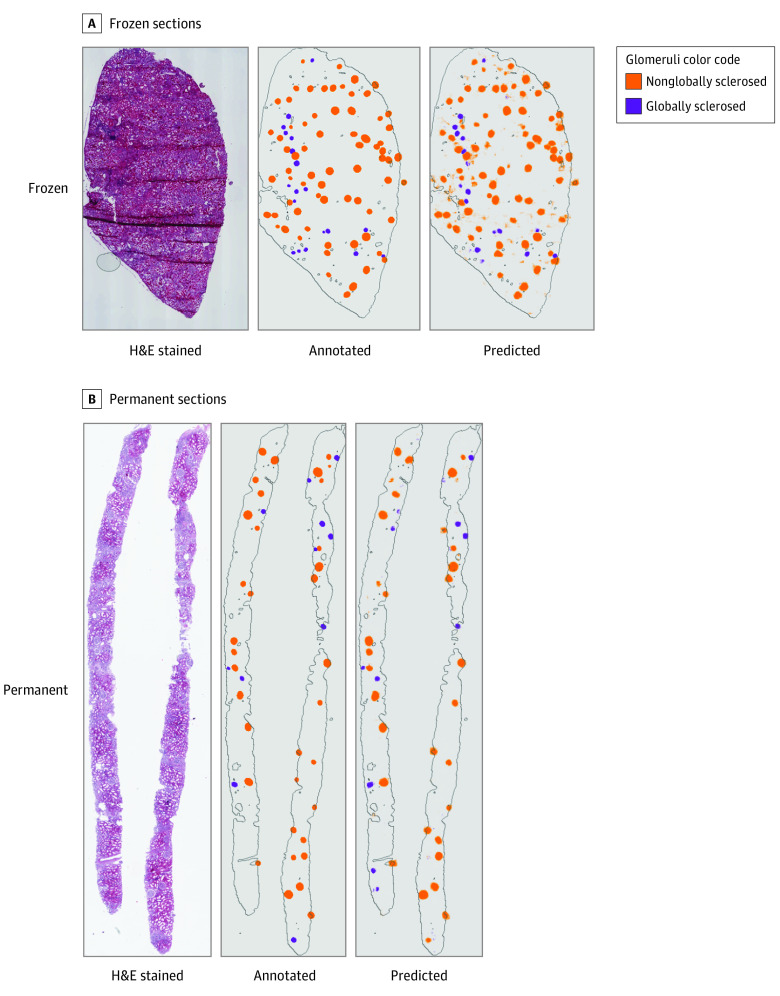
Example Whole-Slide Images With Visualizations of Annotation Ground Truth and Model Prediction Left panels, example hematoxylin-eosin (H&E)–stained whole-slide images. Middle panels, pathologist glomeruli annotations. Right panels, cross-validated model predictions obtained using H&E inputs.

Predicted image outputs for frozen-section and permanent-section WSIs showed qualitative agreement with target annotation maps (Figure 1). Aggregate Dice coefficients were 0.784 for nonglobally sclerosed glomeruli and 0.600 for globally sclerosed glomeruli; aggregate intersection over union metrics for the same groups were 0.645 for nonglobally sclerosed glomeruli and 0.429 for globally sclerosed glomeruli. Notably, even frozen sections with substantial artifacts showed qualitative visual agreement between annotation ground truth and predictions (example shown in Figure 1A).

### Evaluation of Percent Global Glomerulosclerosis Based on Individual Slides

**Figure 2.**
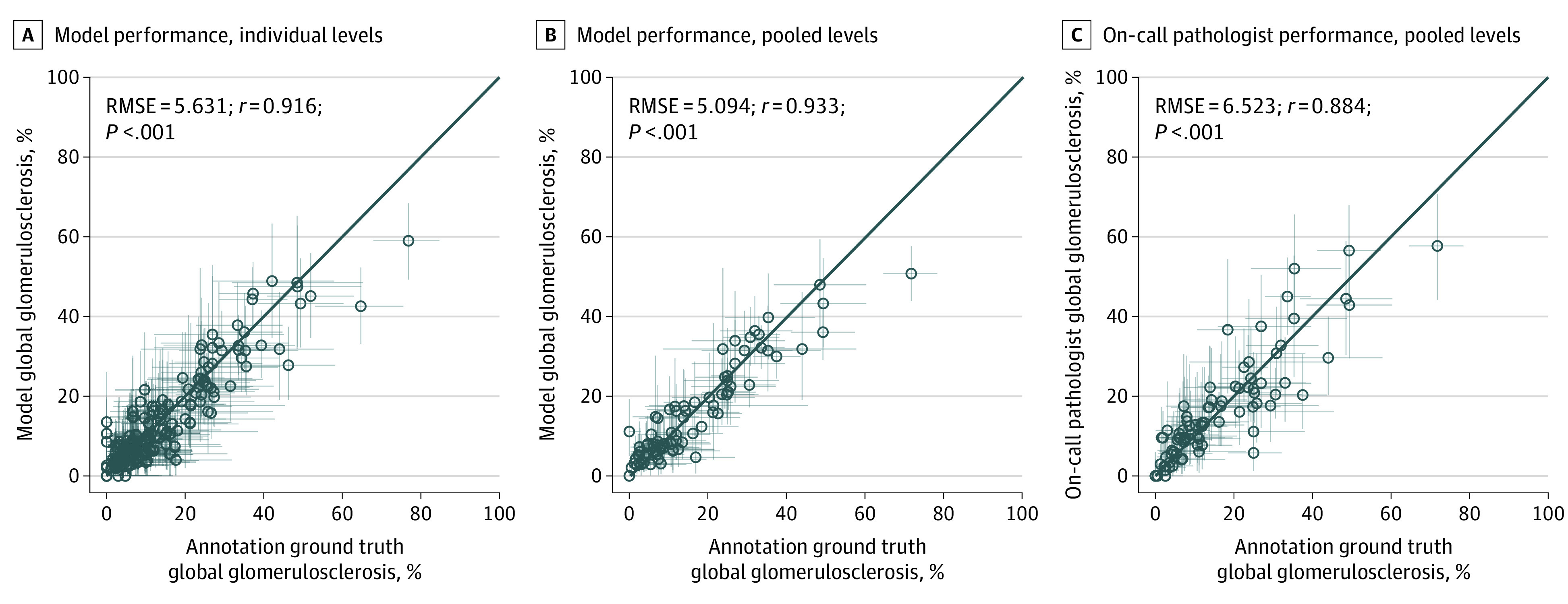
Model and On-Call Pathologists’ Estimates of Percent Global Glomerulosclerosis A, Model predictions of percent global glomerulosclerosis vs expert pathologists’ annotations on individual slide levels, obtained from 10-fold cross-validation. Error bars represent 95% prediction intervals computed from the beta distribution with parameters given by number of globally sclerosed and nonglobally sclerosed glomeruli. B, Same as (A), with results for individual kidneys obtained by pooling glomeruli counts. C, On-call pathologist performance vs expert pathologists’ annotations for corresponding cases. RMSE represents root-mean-square error.

**Figure 3.**
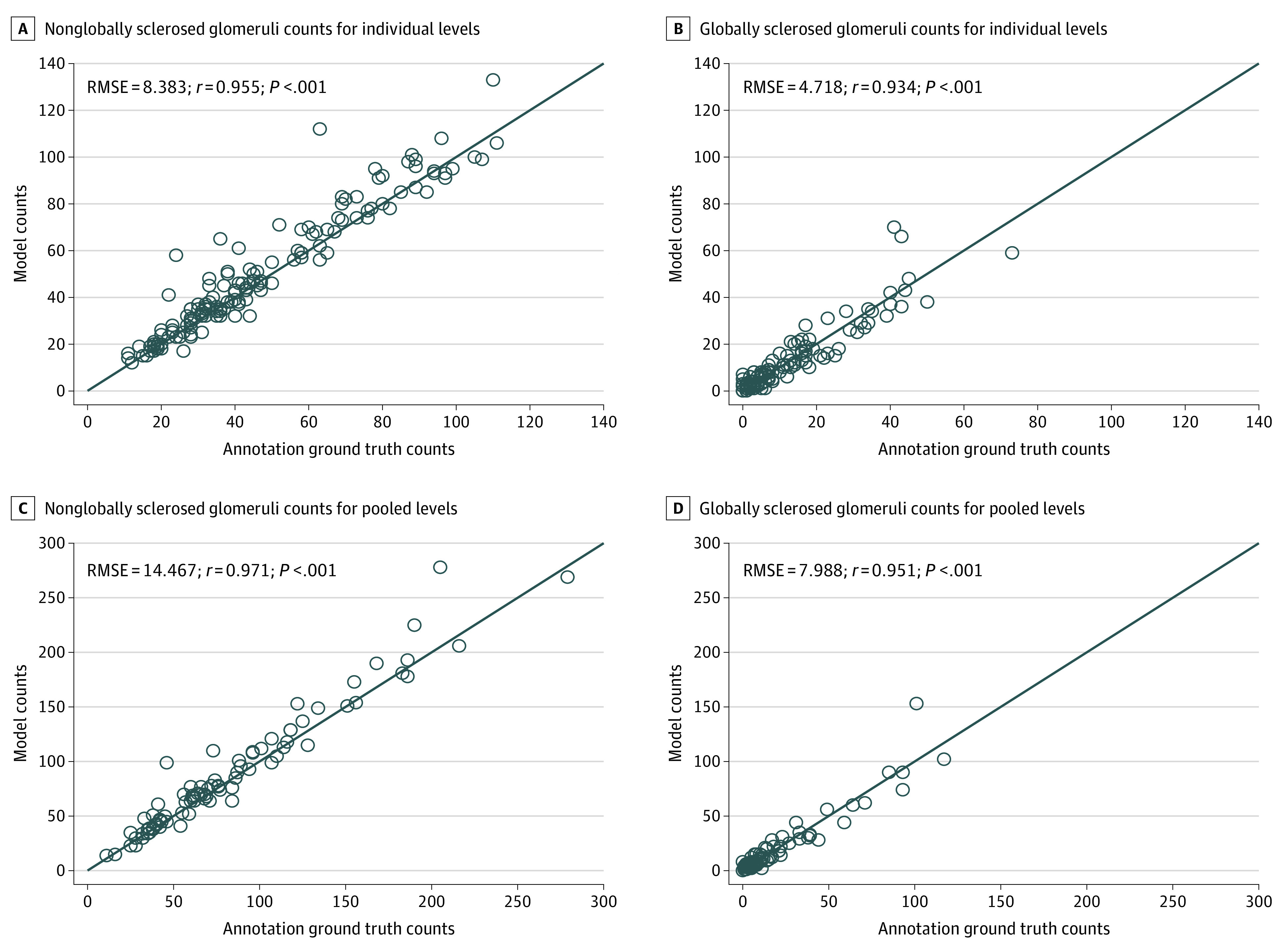
Model Predictions for Number of Glomeruli vs Expert Pathologists’ Annotations RMSE represents root-mean-square error.

Cross-validated glomerulosclerosis predictions on individual slides also exhibited correlation with annotations (*r* = 0.916; 95% CI, 0.886-0.939; and RMSE = 5.631; 95% CI, 4.735-6.517; *P* < .001) (Figure 2A). Separating the results by slide preparation technique indicated that predictions on frozen sections showed similar correlation with ground truth (*r* = 0.918; 95% CI, 0.879-0.944; RMSE = 6.20; *P* < .001) (eFigure 3A in the Supplement), whereas the permanent group showed higher performance (*r* = 0.940; 95% CI, 0.896-0.965; RMSE = 4.32; *P* < .001) (eFigure 3D in the Supplement). The total numbers of glomeruli detected by the model are shown in Figure 3A and B, illustrating the correlations of nonglobally sclerosed glomeruli with ground truth (*r* = 0.955; 95% CI, 0.938-0.967; RMSE = 8.383; *P* < .001) and globally sclerosed glomeruli with ground truth (*r* = 0.934; 95% CI, 0.909-0.952; RMSE = 4.718; *P* < .001). The mean (SD) glomeruli count differences between annotation and prediction were 3.1 (7.8) for nonglobally sclerosed glomeruli and 0.2 (4.7) for globally sclerosed glomeruli. Similar positive results for predicted glomeruli counts were observed when separating slides by treatment (eFigure 2A, B, E, and F in the Supplement).

### Evaluation of Percent Global Glomerulosclerosis Based on Pooled Slides

Pooling levels improved the model’s glomeruli count performance (Figure 3C and D; eFigure 2C, D, G, and H in the Supplement) as well as glomerulosclerosis correlation with annotations, as shown in Figure 2B (*r* = 0.933; 95% CI, 0.898-0.956; and RMSE = 5.094; 95% CI, 3.972-6.301; *P* < .001, for combined frozen and permanent sections), bettering on-call pathologists’ performance on the same cases (*r* = 0.884; 95% CI, 0.825-0.923; and RMSE = 6.523; 95% CI, 5.191-7.783; *P* < .001) (Figure 2C). Global glomerulosclerosis error as measured by RMSE was 22% lower for the model than for on-call pathologists. Concordance between the model’s predictions of global glomerulosclerosis for individual and pooled levels is shown in eFigure 4 in the Supplement as a residual with respect to annotation ground truth.

### Evaluation of Kidney Mischaracterization Risk

**Figure 4.**
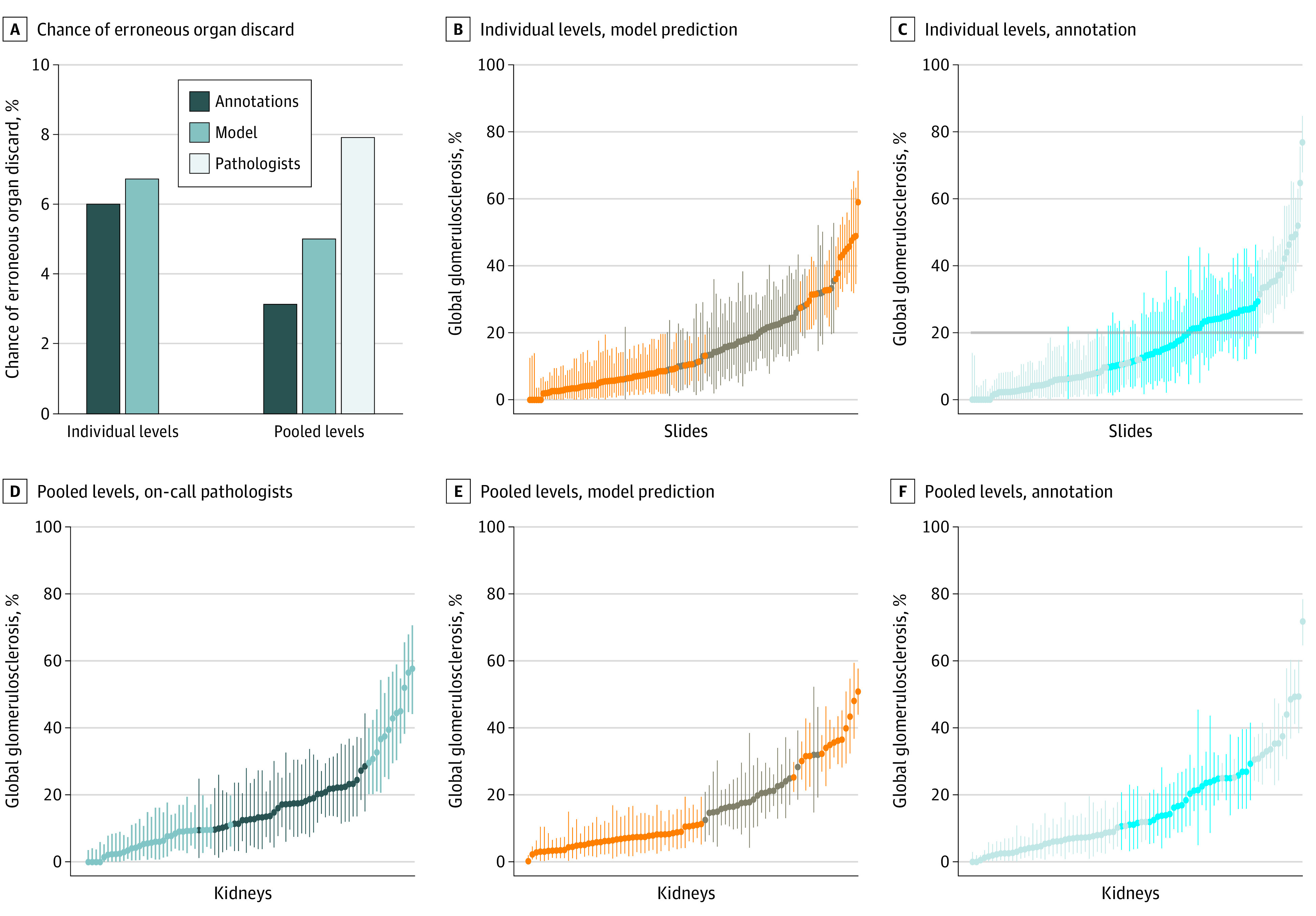
Sorted Percent Global Glomerulosclerosis Estimates in Comparison With Nominal Rejection Cut Point The overall risk of erroneously discarding potentially usable kidneys is shown in panel (A) for evaluations based on individual levels and pooled levels. Results for percent global glomerulosclerosis plotted in increasing order for individual levels derived from model predictions (B) and annotations (C). Results for percent global glomerulosclerosis of individual kidneys obtained from pooling levels derived from on-call pathologist counts (D), model predictions (E), and annotations (F). Error bars represent 95% prediction intervals assuming the measurement is modeled by a beta distribution with parameters given by the number of nonglobally sclerosed and globally sclerosed glomeruli. Nominal organ rejection cut point at 20% global glomerulosclerosis is shown as a horizontal gray line. Cases where the measurement prediction interval crosses the cut point are depicted with darker coloring where there is more than 5% chance of erroneously accepting or rejecting the kidney for transplantation.

Pooled percent global glomerulosclerosis results for the annotations, model predictions, and on-call pathologists were sorted and plotted in order of increasing percent global glomerulosclerosis for all 83 kidneys included in the study, along with corresponding 95% prediction intervals and the 20% cut point for donor organ transplant acceptance or rejection (Figure 4B-F). Because all levels of section are evaluated by the on-call pathologists at the time of biopsy, their results are considered pooled evaluations. Kidneys with prediction intervals overlapping the 20% cut point line are more at risk for erroneous acceptance or rejection if glomeruli counts are incorrectly estimated. The chance of erroneously categorizing a kidney with greater than 20% global glomerulosclerosis is shown in Figure 4A. Using individual slides, the DL model’s projected error rate was 15% lower than for on-call pathologists and nearly identical to ground truth annotations (ie, the ideal case). With pooled levels, the DL model’s projected error rate dropped to 37% lower than that for the on-call pathologists. Similarly, the DL model’s projected error rate for erroneous organ acceptance using individual levels was 21% lower than that for on-call pathologists, and 34% lower when using pooled levels.

The F1 score and Cohen κ showed similar results. The DL model’s F1 score for individual levels having global glomerulosclerosis below 20% was 0.896, and 0.950 for those individual levels above 20%. These metrics improved when pooling levels to 0.926 for those below 20%, and 0.964 for those above 20%. This compared favorably with the F1 scores for on-call pathologists of 0.852 for those below 20%, and 0.929 for those above 20%. Cohen κ for the model predictions on individual levels with respect to ground truth was 0.847, improving to 0.891 for pooled levels. Cohen κ for on-call pathologists with respect to pooled annotations was lower, at a value of 0.781, and was 0.714 when compared with the model’s pooled level predictions. Concordance between pathologist and model results for pooled level results is shown in eFigure 5 in the Supplement as a residual with respect to ground truth, sorted by ground truth global glomerulosclerosis percentage and total glomeruli count.

**Figure 5.**
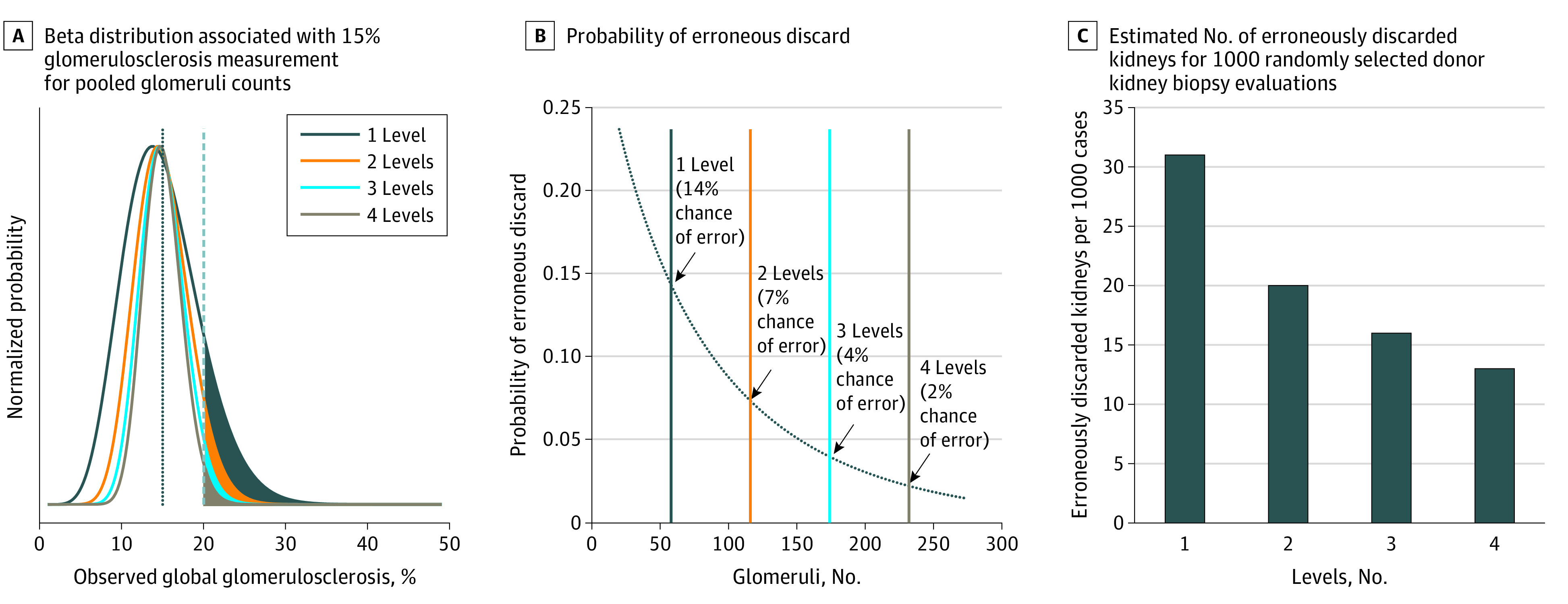
Global Glomerulosclerosis Measurement Distribution Modeling for Estimating Probability of Erroneous Kidney Discard A, The beta distribution uses the mean number of glomeruli per level (58) observed in this study. Nominal 20% rejection cut point shown by vertical dashed gray line. The chance of erroneous organ rejection is proportional to the area under the curve to the right of the cut point line. B, Probability of erroneous discard for curves shown in A. C, Estimations use on-call pathologists’ glomeruli counts as a surrogate for ground truth. The number of erroneous discards is halved by reading 4 levels vs only 1 level.

The value of multilevel examination is shown by evaluating the prediction intervals from the beta distribution. An illustration of the beta distribution for a hypothetical biopsy specimen with 15% global glomerulosclerosis is shown in Figure 5A for pools of 1, 2, 3, and 4 levels, assuming each level has 58 observed glomeruli (the mean number for this study). The height of each curve at a given value on the horizontal axis can be interpreted as the relative likelihood of estimating percent global glomerulosclerosis to be that value, given the true distribution of sclerosed and normal glomeruli. The area under the curve thus yields an estimate of the likelihood of obtaining global glomerulosclerosis estimates within the limits of integration. The distribution narrowed with increased pooling. More importantly, the normalized area under the curve beyond the nominal 20% rejection cut point decreased from 14% using only a single level to 2% when pooling 4 levels (Figure 5B), a 7-fold decrease in the chance for incorrectly overestimating global glomerulosclerosis and erroneously discarding what should be a useable organ.

To further illustrate the benefits of level pooling, glomeruli counts for 1000 randomly selected evaluations of donor biopsy specimens (from the same database as the 83 kidney biopsy specimens used in this study) were used to simulate the effects of level pooling for a large population. The on-call pathologist estimates were used as surrogates for ground truth glomeruli counts, and data pooling was simulated by multiplying the reported counts per level by the number of simulated levels in the pool. Applying the analysis described above to this scenario, the number of erroneous organ discards for every 1000 kidneys would decrease from 31 to 13 by increasing the number of levels evaluated from 1 to 4 (Figure 5C).

As a demonstration of the potential clinical workflow with the incorporation of DL techniques, the DL model’s predicted annotations for 25 cases from the study data set were randomly selected (5 each with 0%-5%, 6%-10%, 11%-15%, 16%-20%, and >20% global glomerulosclerosis) and were submitted to a pathologist, who evaluated the histology images with overlaid model-generated glomeruli classifications. The pathologist then corrected any missed or inaccurately labeled glomeruli in a manner and time frame consistent with current clinical practice. The pathologist-amended evaluation was better correlated with ground truth (*r* = 0.958) and had lower error (RMSE = 4.352) than either the on-call pathologist (*r* = 0.613; RMSE = 0.898) or the DL model alone (*r* = 0.847; RMSE = 7.535) (eFigure 7 in the Supplement).

## Discussion

The DL model produced encouraging results, in both qualitative (visual) and quantitative findings, and recapitulated results described in earlier work by members of our group on a smaller training set.^34^ The model performed well using either frozen sections or permanent sections, bettering on-call pathologists’ performance. The time for the model to process an individual WSI was approximately 5 minutes, well within the typical constraints of a pathology intraoperative consultation.

Magnification of counting errors when using a small sample highlights the value gained from pooling results from multiple levels obtained from a single kidney biopsy. The typical thickness of a donor kidney biopsy specimen is 1 mm. The pathologist only examines a representative 5-μm–thick section of this tissue, leaving a substantial portion of unevaluated kidney unexamined. Although glomeruli sampled in subsequent sections may not be independent, the slide preparation process can lead to substantial section-to-section variability in global glomerulosclerosis, irrespective of observer variability (eFigure 6 in the Supplement). By evaluating more tissue sections, the effect of this variability can be minimized and the reliability of the biopsy specimen evaluation enhanced. This benefit is clearly observed in the present study for each metric (Figures 2-4), which all exhibited improvement with examination of additional tissue.

Current standard of care requires evaluation of only 25 glomeruli and 1 to 2 levels of section because more evaluations are not practically achievable by human pathologists in the time-sensitive context of organ transplant. The use of DL techniques to augment human capability in this setting could add vitally needed organs to the donor pool. A potential clinical workflow with the incorporation of DL techniques could be as follows: a specimen arrives in the frozen-section laboratory, where a frozen-section slide is prepared and scanned. The WSI is then uploaded to a secure location for analysis using the DL model. While the DL model is analyzing, the pathologist may log in and review the sample for other pertinent findings. The DL model result would be available within 5 to 10 minutes, presented to the pathologist as a graphical overlay of glomeruli classifications on the histology image, then verified (and amended if necessary) by the pathologist, and incorporated into the report. The actual report would directly interface with the clinical electronic health record.

### Limitations

There are some limitations to this study. It was a single-center study. Although the WSIs were generated using 2 scanners at 2 institutions, the frozen-section data set was entirely generated at 1 institution, whereas the permanent-section data set was generated from another. Although a small preliminary data set (n = 17) suggested that model predictions on frozen sections exhibited reasonable correspondence with associated permanent sections and that the model outperformed on-call pathologists on these frozen sections (eFigure 8 in the Supplement), this study did not directly address the separate issue of how closely the frozen sections (as well as pathologists’ evaluations of them) corresponded to permanent sections subsequently acquired and processed from the same biopsy specimen.

The data set was small compared with other DL studies. However, nearly 8500 glomeruli were examined in total, a relatively high number. The limitation in evaluating greater numbers of cases lies in the time-consuming process of serially annotating WSIs. To further evaluate the robustness of this model, additional studies will be required wherein the model is tested using WSIs generated from additional laboratories and scanners.

## Conclusions

This prognostic study found better performance for quantifying percent global glomerulosclerosis from WSIs of frozen and of permanent hematoxylin-eosin–stained donor transplant kidney biopsy specimens by a DL model than by on-call board-certified pathologists. Performance was further improved by examining additional tissue sections, a process that is beyond the capacity of pathologists in the time-sensitive nature of evaluating donor biopsy specimens. The results indicated decreased likelihood of mischaracterizing percent global glomerulosclerosis when using the DL model, thereby decreasing the likelihood of inappropriate donor organ discard or of using an organ that is suboptimal. The findings illustrate the substantial gains that could be realized using DL methods in surgical pathology clinical practice.

## Notes

Supplement. **eFigure 1.** Change in Expert Pathologist Annotations With Each Annotation Revision **eFigure 2.** Model and On-Call Pathologists’ Estimates of Percent Global Glomerulosclerosis Separated by Slide Preparation: Frozen (Top) or Permanent (Bottom) **eFigure 3.** Model Predictions for Number of Glomeruli vs Expert Pathologists’ Annotations Separated by Slide Preparation: Frozen (Top) or Permanent (Bottom) **eFigure 4.** Concordance Between the Model’s Predictions of Global Glomerulosclerosis for Individual and Pooled Levels, Shown as a Residual With Respect to Annotation Ground Truth **eFigure 5.** Concordance Between Model Predictions and On-Call Pathologists’ Report for Global Glomerulosclerosis on Individual Kidneys Using Pooled Levels, Shown as Residuals With Respect to Ground Truth Glomerulosclerosis **eFigure 6.** Variability of Percent Global Glomerulosclerosis for Biopsies With Multiple Levels of Section **eFigure 7.** Pathologist Reevaluation of DL Model Predictions Improves Estimate of Percent Global Glomerulosclerosis **eFigure 8.** Correlation of Model and On-Call Pathologist Assessment of Percent Global Glomerulosclerosis for Corresponding Frozen and Permanent Sections
