# Supplementary material for: Development and Validation of a Deep Learning Model to Quantify Glomerulosclerosis in Kidney Biopsy Specimens
Source: JAMA Netw Open. 2021 Jan 20;4(1):e2030939. doi: 10.1001/jamanetworkopen.2020.30939 (PMC7818108; doi:10.1001/jamanetworkopen.2020.30939)
Supplement: Supplement. — eFigure 1. Change in Expert Pathologist Annotations With Each Annotation Revision eFigure 2. Model and On-Call Pathologists’ Estimates of Percent Global Glomerulosclerosis Separated by Slide Preparation: Frozen (Top) or Permanent (Bottom) eFigure 3. Model Predictions for Number of Glomeruli vs Expert Pathologists’ Annotations Separated by Slide Preparation: Frozen (Top) or Permanent (Bottom) eFigure 4. Concordance Between the Model’s Predictions of Global Glomerulosclerosis for Individual and Pooled Levels, Shown as a Residual With Respect to Annotation Ground Truth eFigure 5. Concordance Between Model Predictions and On-Call Pathologists’ Report for Global Glomerulosclerosis on Individual Kidneys Using Pooled Levels, Shown as Residuals With Respect to Ground Truth Glomerulosclerosis eFigure 6. Variability of Percent Global Glomerulosclerosis for Biopsies With Multiple Levels of Section eFigure 7. Pathologist Reevaluation of DL Model Predictions Improves Estimate of Percent Global Glomerulosclerosis eFigure 8. Correlation of Model and On-Call Pathologist Assessment of Percent Global Glomerulosclerosis for Corresponding Frozen and Permanent Sections [file jamanetwopen-e2030939-s001.pdf]

Supplementary Online Content

Marsh JN, Liu TC, Wilson PC, Swamidass SJ, Gaut JP. Development and validation of a deep learning model to quantify glomerulosclerosis in kidney biopsy specimens. *JAMA Netw Open*. 2021;4(1):e2030939. doi:10.1001/jamanetworkopen.2020.30939

eFigure 1. Change in Expert Pathologist Annotations With Each Annotation Revision

eFigure 2. Model and On-Call Pathologists' Estimates of Percent Global Glomerulosclerosis Separated by Slide Preparation: Frozen (Top) or Permanent (Bottom)

eFigure 3. Model Predictions for Number of Glomeruli vs Expert Pathologists' Annotations Separated by Slide Preparation: Frozen (Top) or Permanent (Bottom)

eFigure 4. Concordance Between the Model's Predictions of Global Glomerulosclerosis for Individual and Pooled Levels, Shown as a Residual With Respect to Annotation Ground Truth

eFigure 5. Concordance Between Model Predictions and On-Call Pathologists' Report for Global Glomerulosclerosis on Individual Kidneys Using Pooled Levels, Shown as Residuals With Respect to Ground Truth Glomerulosclerosis

eFigure 6. Variability of Percent Global Glomerulosclerosis for Biopsies With Multiple Levels of Section

eFigure 7. Pathologist Reevaluation of DL Model Predictions Improves Estimate of Percent Global Glomerulosclerosis

eFigure 8. Correlation of Model and On-Call Pathologist Assessment of Percent Global Glomerulosclerosis for Corresponding Frozen and Permanent Sections

This supplementary material has been provided by the authors to give readers additional information about their work.

© 2021 Marsh JN et al. *JAMA Network Open*.

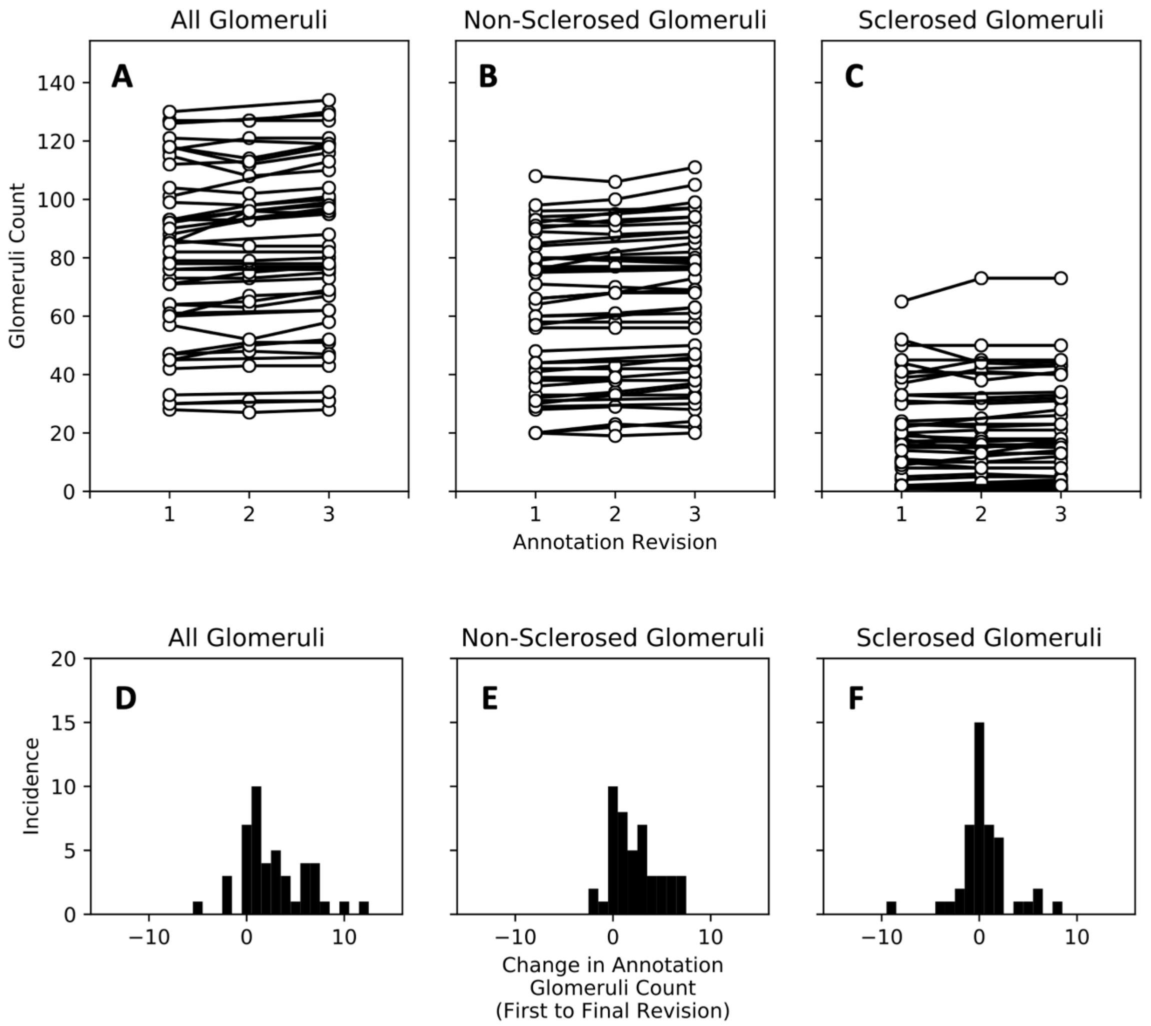

eFigure 1. Change in Expert Pathologist Annotations With Each Annotation Revision

A) Total number of non-globally sclerosed and globally sclerosed glomeruli for a subset of slide annotations for 1st, 2nd, and 3rd annotation revision. B,C) Same as A, separated into non-globally sclerosed glomeruli and globally sclerosed glomeruli, respectively. D) Distribution of glomeruli count difference between 1st and 3rd annotation revision for all annotated glomeruli. E,F) Same as D, separated into non-globally sclerosed glomeruli and globally sclerosed glomeruli, respectively.

© 2021 Marsh JN et al. *JAMA Network Open*.

eFigure 2. Model and On-Call Pathologists' Estimates of Percent Global Glomerulosclerosis Separated by Slide Preparation: Frozen (Top) or Permanent (Bottom)

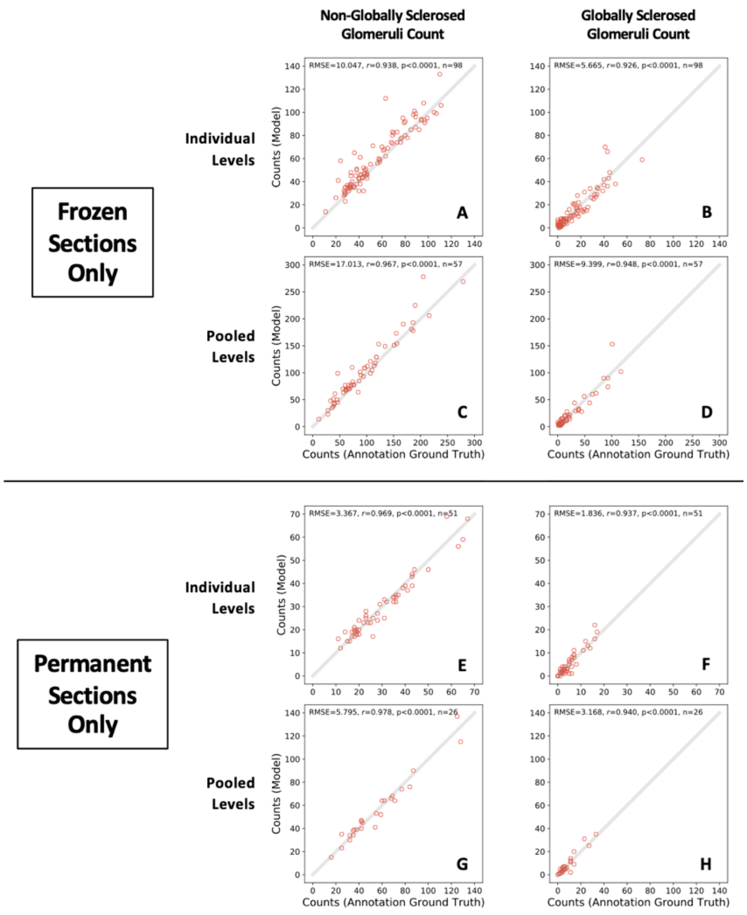

A, D) Model predictions of percent global glomerulosclerosis vs expert pathologists' annotations on individual frozen section slide levels, obtained from 10-fold cross-validation. Error bars represent 95% prediction intervals computed from beta distribution with parameters given by number of globally sclerosed and non-globally sclerosed glomeruli. B, E) Same as A, with results for individual kidneys obtained by pooling glomeruli counts. C, F) On-call pathologist performance vs expert pathologists' annotations for corresponding cases.

© 2021 Marsh JN et al. *JAMA Network Open*.

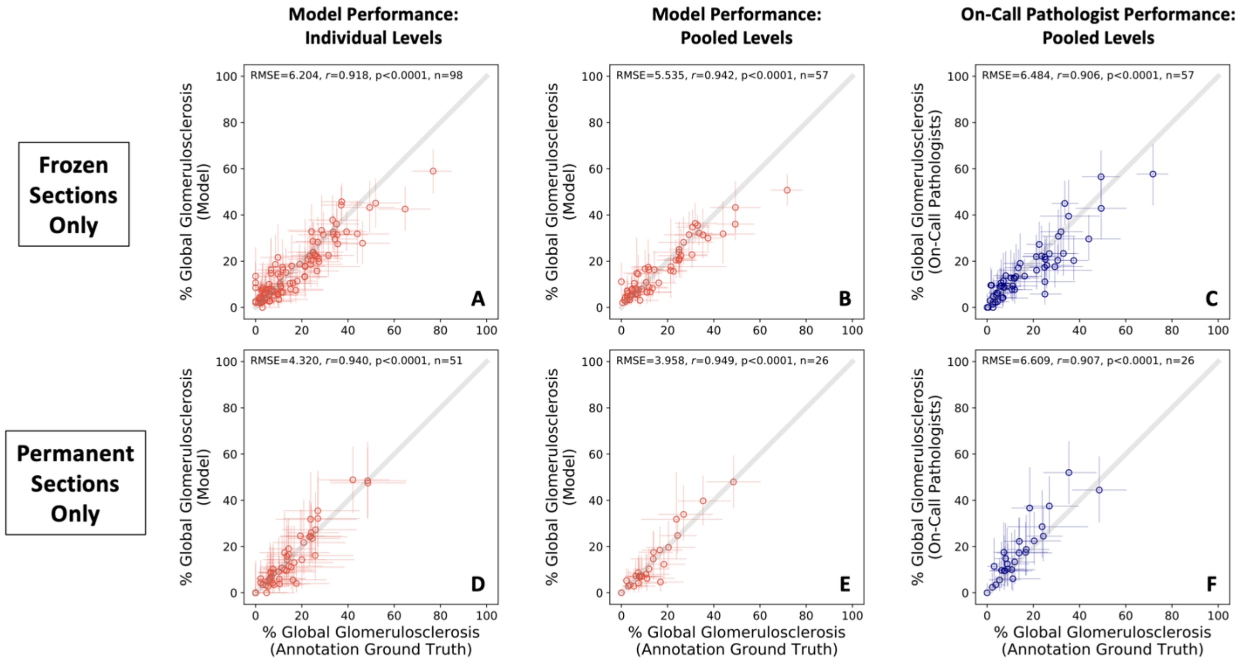

eFigure 3. Model Predictions for Number of Glomeruli vs Expert Pathologists' Annotations Separated by Slide Preparation: Frozen (Top) or Permanent (Bottom)

A, E) Non-globally sclerosed glomeruli counts for individual levels. B, F) Globally sclerosed glomeruli counts for individual levels. C, G) Non-globally sclerosed glomeruli counts for individual kidneys obtained by pooling levels. D, H) Globally sclerosed glomeruli counts for individual kidneys obtained by pooling levels.

© 2021 Marsh JN et al. *JAMA Network Open*.

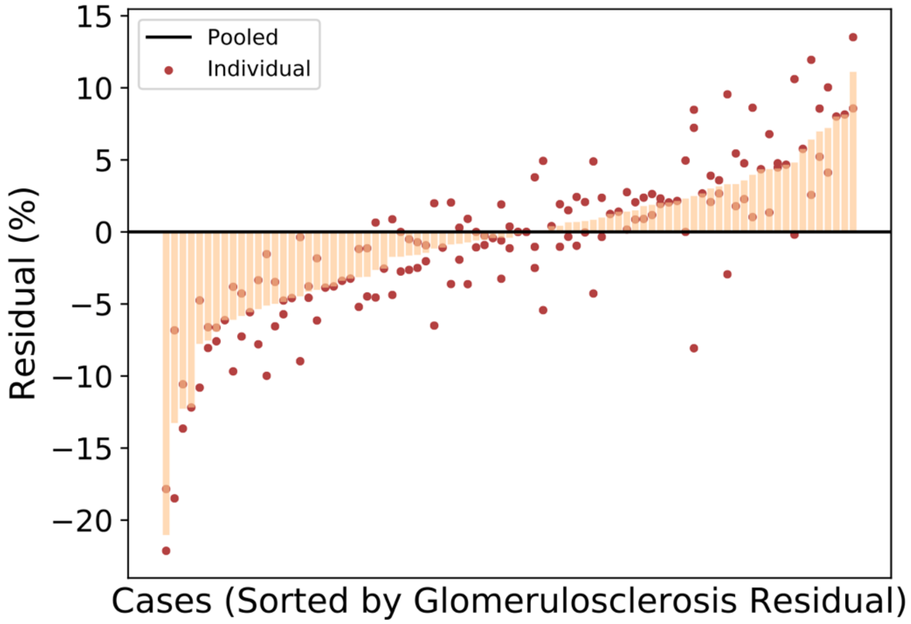

eFigure 4. Concordance Between the Model's Predictions of Global Glomerulosclerosis for Individual and Pooled Levels, Shown as a Residual With Respect to Annotation Ground Truth

Bars represent pooled residuals, and overlaid dots represent individual residuals for each respective kidney. Results are displayed in order of increasing pooled residual.

© 2021 Marsh JN et al. *JAMA Network Open*.

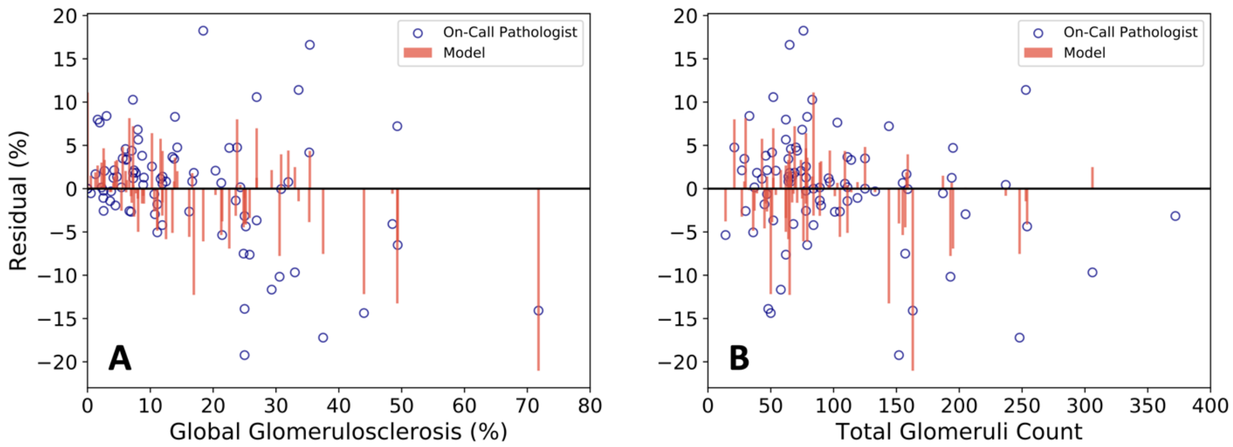

eFigure 5. Concordance Between Model Predictions and On-Call Pathologists' Report for Global Glomerulosclerosis on Individual Kidneys Using Pooled Levels, Shown as Residuals With Respect to Ground Truth Glomerulosclerosis

A) Paired bars show residuals for model (red) and on-call pathologists (blue) as a function of ground truth global glomerulosclerosis. B) Same as A), but displayed in order of increasing total pooled glomeruli count.

© 2021 Marsh JN et al. *JAMA Network Open*.

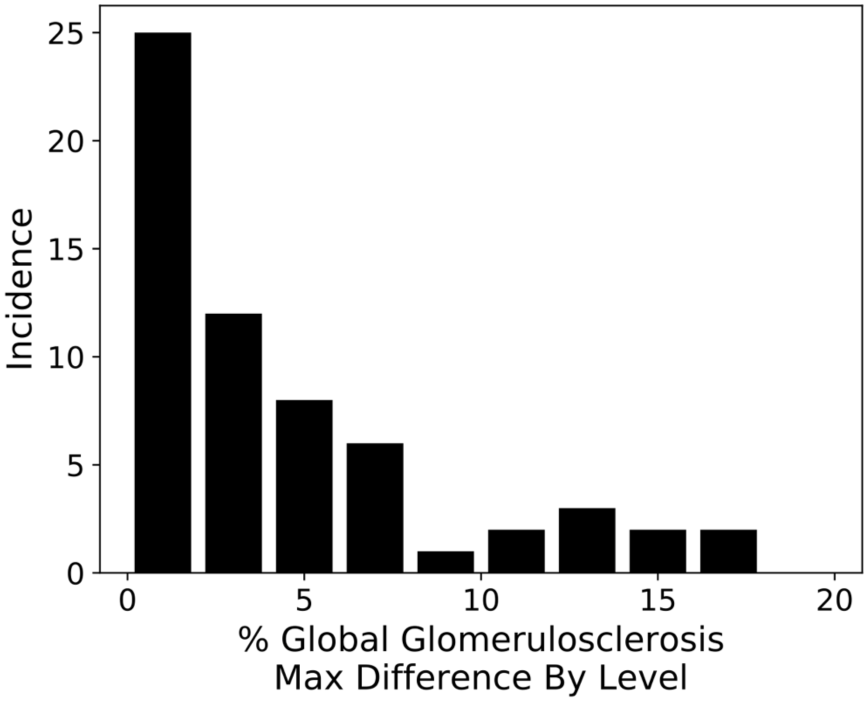

eFigure 6. Variability of Percent Global Glomerulosclerosis for Biopsies With Multiple Levels of Section

The range of global glomerulosclerosis values for each kidney was computed as the absolute difference between the maximum and minimum percent global glomerulosclerosis of all levels of section and shown here as a histogram, to indicate variability associated with tissue processing. Percent global glomerulosclerosis values were derived from ground truth annotations.

© 2021 Marsh JN et al. *JAMA Network Open*.

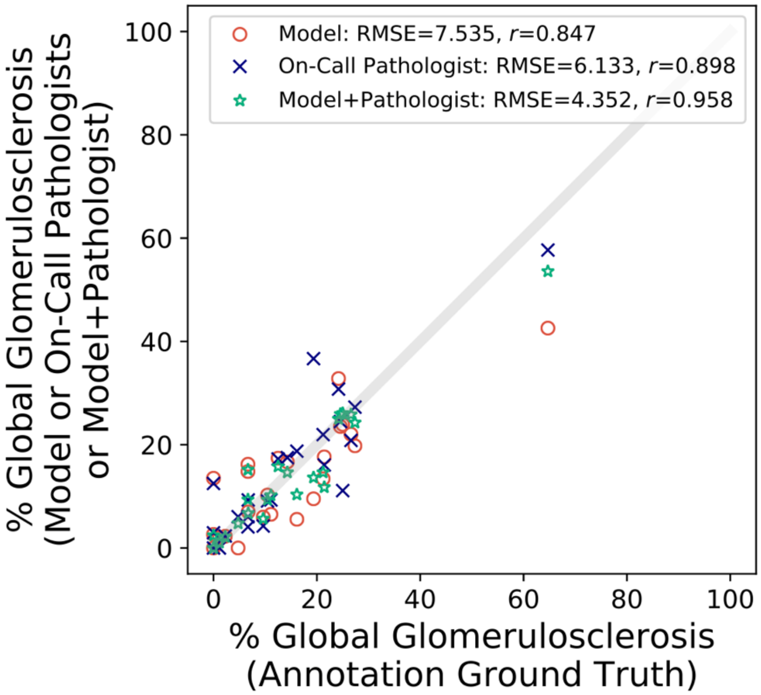

eFigure 7. Pathologist Reevaluation of DL Model Predictions Improves Estimate of Percent Global Glomerulosclerosis

Glomeruli classification results for a subset of 25 cases examined in this study were presented to a pathologist for verification and correction (if needed). The resulting estimates computed for percent global glomerulosclerosis (green stars) more closely matched ground truth annotations than either on-call pathologists (blue crosses) or the DL model (orange circles) alone.

© 2021 Marsh JN et al. *JAMA Network Open*.

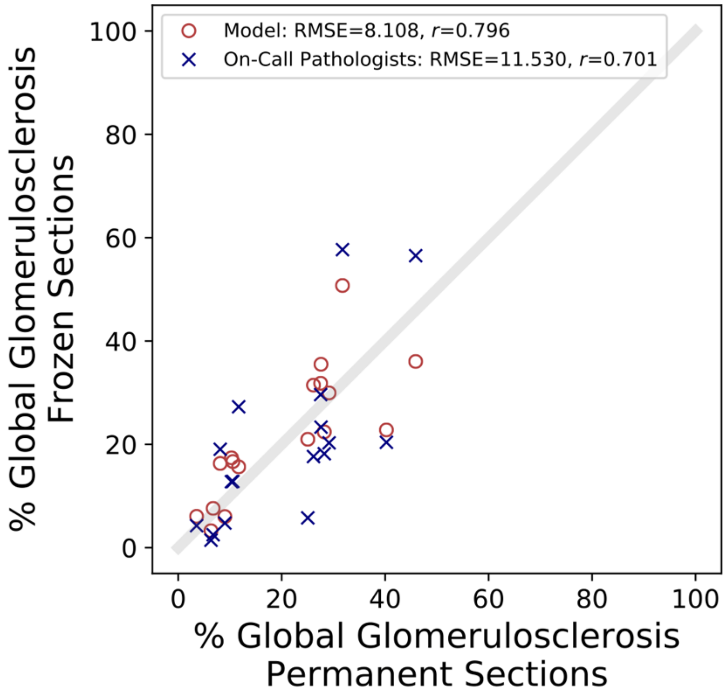

eFigure 8. Correlation of Model and On-Call Pathologist Assessment of Percent Global Glomerulosclerosis for Corresponding Frozen and Permanent Sections

Percent global glomerulosclerosis is shown for 17 kidneys with both frozen and permanent sections. DL model predictions (orange circles) and on-call pathologist estimates (blue crosses) are shown on y-axis, correlated with model predictions for corresponding permanent sections on x-axis. Correlation and RMSE values are better for the model than on-call pathologists in this preliminary dataset.

© 2021 Marsh JN et al. *JAMA Network Open*.
